# HIV immunological nonresponders show low SKAP1 concentration and DNA hypermethylation in the SKAP1 promotor region

**DOI:** 10.1097/QAD.0000000000004529

**Published:** 2026-05-11

**Authors:** Wilhelm A.J.W. Vos, Suzanne D.E. Ruijten, Xun Jiang, Nienke van Unen, Willem van Snippenberg, Albert L. Groenendijk, Marc J.T. Blaauw, Louise E. van Eekeren, Nadira Vadaq, Hanneke Maas, Malin Östman, Maartje C.P. Cleophas, Kees Brinkman, Jan van Lunzen, Willem L. Blok, Janneke E. Stalenhoef, Yang Li, Mihai G. Netea, Linos Vandekerckhove, Jéssica C. dos Santos, Vasiliki Matzaraki, Cheng-Jian Xu, Andre J.A.M. van der Ven

**Affiliations:** a Department of Internal Medicine, Radboud University Medical Center, Nijmegen; b Department of Internal Medicine and Infectious Diseases, OLVG, Amsterdam, The Netherlands; c Centre for Individualized Infection Medicine; d TWINCORE, a joint venture between the Helmholtz-Centre for Infection Research (HZI) and the Hannover Medical School (MHH), Hannover, Germany; e HIV Cure Research Centre, Department of Internal Medicine and Pediatrics, Ghent University, Ghent, Belgium; f Department of Internal Medicine and Department of Medical Microbiology and Infectious diseases, ErasmusMC, Erasmus University, Rotterdam, The Netherlands; g Department of Internal Medicine and Infectious Diseases, Elizabeth-Tweesteden Ziekenhuis; h Department of Immunology and Metabolism, Life and Medical Sciences Institute, University of Bonn, Bonn, Germany.

**Keywords:** DNA methylation, immunological nonresponders, single-nucleotide polymorphisms, Src kinase-associated phosphoprotein 1

## Abstract

**Objective:**

The aim of this study was to improve understanding of biological pathways underlying inadequate CD4^+^ T-cell restoration after initiating antiretroviral treatment as these so called Immunological nonresponders are at increased risk for morbidity and mortality while treatment options are lacking.

**Design:**

We compared baseline multiomics data from 88 Immunological nonresponders and 1467 immunological responders that participated in the 2000HIV study, separated into a discovery and validation cohort.

**Methods:**

We measured expression levels of 2367 plasma proteins, comparing the immunological responders and nonresponders. As this highlighted low Src kinase-associated phosphoprotein 1 (SKAP1) levels in Immunological nonresponders, we measured intracellular SKAP1 levels in CD4^+^ T-cells, investigated DNA methylation and assessed single-nucleotide polymorphisms (SNPs). We also explored whether HIV or CMV infection may influence SKAP1 expression.

**Results:**

SKAP1 plasma levels were significantly lower in Immunological nonresponders in both cohorts. SKAP1 plasma concentrations reflected intracellular levels in CD4^+^ T-cells. DNA methylation analysis showed significant hypermethylation at the *SKAP1* promotor region. Three SNPs close to the *SKAP1* gene were associated with poor Immunological response. Preliminary data suggest that HIV or CMV may influence SKAP1 levels.

**Conclusion:**

Our data show decreased SKAP1 concentrations in immunological nonresponders, potentially driven by hypermethylation of the *SKAP1* promoter. Downregulation of SKAP1, which is known to play a role in T cell proliferation and migration, may therefore contribute to the poor restoration of CD4^+^ cell count after ART.

## Introduction

The long-term prognosis of people with HIV (PWH) was dramatically improved by the introduction of combination antiretroviral therapy (cART). cART inhibits viral replication and induces immune restoration as observed through undetectable viral load and rise in CD4^+^ T cell numbers, respectively. However in 10–40% of PWH, depending on the definition used, cART insufficiently restores CD4^+^ T cell numbers despite adequate viral suppression. These individuals, known as immunological nonresponders (INR), are at increased risk of morbidity and mortality, compared to immunological responders (IR) [1,2].

Several interventions for INR have been attempted, including cART intensification and interleukin (IL)-2 administration, but none improved the long-term clinical outcomes for INR [3–5]. Some of these interventions are based on current concepts of the pathogenesis of INR that include decreased generation of CD4^+^ T cells through impaired hematopoiesis and thymic output, enhanced apoptosis, lymphoid tissue fibrosis, increased microbial gut translocation, elevated systemic inflammation, persistent viral replication, and immune exhaustion [1,6,7]. The possible favorable effects of IL-7 substitution, which improves thymopoiesis, peripheral proliferation and survival of mature T cells, has been obscured by its role in expanding the HIV reservoir [8,9]. Despite these efforts, no therapeutic interventions for INR are presently available.

High-throughput omics technologies are increasingly being used to better understand pathophysiological processes. These encompass targeted proteomic profiling that aims to identify the expression of a wide range of plasma proteins including intracellular, secreted, and low-abundance proteins [10].

The primary aim of the present study was to identify pathways related to the INR phenotype that may reveal new therapeutical targets. Therefore we used extensive plasma proteomics comparing INR and IR, followed by -omics data layers analysis and in-vitro experiments. Out of 2367 plasma proteins, only Src kinase-associated phosphoprotein 1 (SKAP1) concentrations were significantly and consistently lower in INR. Subsequently, the relation between low plasma SKAP1 and low SKAP1 expression in CD4^+^ T cell was confirmed, as well as hypermethylation of the *SKAP1* promoter region as a factor contributing to low SKAP1 levels in INR.

## Materials and methods

### Study population and data collection

The 2000HIV study (clinical trials NTC03994835) is a multicenter longitudinal cohort study of 1895 virally suppressed PWH on suppressive cART enrolled in four HIV treatment centers in the Netherlands, separated into an independent discovery (*n* = 1559) and validation (*n* = 336) cohort, as described previously [11]. Inclusion criteria entailed ≥18 years of age, HIV-1 positivity, ≥6 months on cART and latest viral load < 200 copies/ml. Exclusion criteria were pregnancy or current signs of active infection. After informed consent, clinical data were collected through questionnaires and medical examination, and EDTA blood was sampled after ≥4 h of fasting. Participants who spontaneously controlled HIV without cART or use immunomodulatory drugs were excluded.

In line with literature, INR were defined as CD4^+^ T cell <350/μl after ≥2 years of suppressive cART [12]. In contrast, IR were PWH with CD4^+^ T cell >500/μl under suppressive cART, as CD4^+^ T cell >500/μl is associated with normal life expectancy [13].

### Ethics

The 2000HIV study protocol was approved by the accredited medical research ethics committee Arnhem-Nijmegen (NL68056.091.81). Informed consent was obtained from all participants. The principles of the Declaration of Helsinki were followed.

### Plasma proteomics

#### Proteomics measurement

Plasma proteomics were measured through Olink Proteomics AB (Uppsala Sweden) using a commercially available proximity extension assay (PEA) in three batches. In total, 3072 plasma proteins were measured (Olink explore panel) divided over inflammatory-, oncology-, cardiometabolic-, and neurology-related panels. In each panel IL6, TNF, CXCL8, LMOD1, SCRIB and IDO1 were measured as technical duplicates to maintain quality control. Values used in calculations are Normalized Protein Expression values (NPX values) and are the result of quality control by Olink known as Olink PEA QC process. In brief, NPX values are normalized values over different plates through subtraction of values for extension control and plate control. A correction factor is used to cancel out any background noise. We excluded proteins that were detected in <75% of samples (*n* = 547). We performed quality control per sample using principal component analysis (PCA) to identify and exclude sample outliers above or below four standard deviations from the mean principal component (PC) 1 or PC2 (*n* = 7). Finally, 2367 proteins in 1777 samples were eligible for analysis.

#### Statistical analysis of plasma proteomics

To identify potential confounders, we assessed associations between the first five PCs of plasma proteomic concentrations and potential confounders using a linear regression model in the discovery cohort. The results were presented as adjusted *R*^2^ values, with variables having high adjusted R^2^ values considered as potential confounders (Supplementary Figure 1A, B, Supplemental Digital Content). This process identified age, sex at birth (sex), seasonality, BMI and current smoking status as significant confounders in the discovery cohort. Seasonality was modeled using a sine and cosine wave with a period of 365.25 days. Together, these two terms can form a sine wave with any phase with a frequency of a year [14]. The list of identified confounders in the discovery cohort was also applied to the validation cohort.

For plasma protein concentrations we used differential expression analysis using the R Limma package to determine and visualize differentially expressed proteins corrected for age, sex, seasonality, BMI and current smoking status.

### Intracellular Src kinase-associated phosphoprotein 1 measurement in CD4^+^ T cells

PBMCs of five INR with the lowest and eight IR with the highest SKAP1 concentrations in the plasma proteomics obtained during the baseline visit of the 2000HIV study were used to measure SKAP1 levels in CD4^+^ T cells. After CD4^+^ T cell isolation, intracellular detection of SKAP1 levels was measured using Western Blot (see supplement information 1, Supplemental Digital Content for more details). Intracellular SKAP1 concentrations of the high and low plasma SKAP1 participants were compared using Mann–Whitney *U* test. *P* < 0.05 was considered significant.

### Epigenetics DNA methylation

#### DNA methylation assay

DNA extraction from EDTA whole blood was performed using an automated system, the ChemagicStar configuration. This setup includes the Microlab STAR and Chemagen Magnetic Separation Module 1 from Hamilton Robotics. The extraction method utilizes Chemagen nucleic acid extraction technology with magnetic polyvinyl alcohol (M-PVA) beads, following a standard automated bind-wash-elute procedure. Subsequently, DNA concentration and its 260/280 nm ratio were determined using a NanoDrop spectrophotometer. Following this, samples were normalized to a concentration of 50 ng/μl in TE-buffer and randomly distributed across plates.

High-quality DNA samples were then chosen for genome-wide DNA methylation profiling using the Illumina Infinium MethylationEPIC BeadChip array (MethylEPIC v1 manifest B5). Rigorous quality control measures were applied at both the sample and probe levels. Each subject was analyzed separately. Methylation values were derived from the raw IDAT files using the minfi package in R (v.4.2.0) [15–17]. Preprocessing steps involved the exclusion of two gender mismatch samples from the discovery cohort and one low-quality sample from the validation cohort (call rate < 99%). Probes with methylation values missing in over 10% of samples and those located within the sex chromosome were also eliminated. Furthermore, probes containing SNPs at the target CpG sites with a minor allele frequency >5% in European populations and probes mapping to multiple loci were removed. Stratified quantile normalization was applied [18], and methylation values were utilized to estimate the proportions of six immune cell types.

#### Statistical analysis of DNA methylation

For the analysis of DNA methylation, a robust approach was employed to address extreme outliers by trimming the methylation dataset. This involved using the 25th percentile − 3 × inter quartile range (IQR) and 75th percentile + 3 × IQR method.

To identify associations between differentially methylated CpG sites and INR, a linear regression model was employed. The model utilized the methylation *M* value as the first input variable and INR as the outcome variable. In the discovery cohort, the model was adjusted for various covariates, including age, sex, BMI, technical covariates, seasonality and proportions of immune cells. CD4^+^ T cell count was excluded from this as INR are defined by their CD4^+^ T cell count. Additionally, for epigenome-wide association studies (EWAS) encompassing all ethnicities, the model incorporated the top five principal components extracted from the genotype data. This inclusion aimed to account for ethnic variations and correct for potential confounding effects related to genetic ancestry.

In the validation cohort, due to the small sample size (INR = 26), to avoid the numerical instability issues, the proportions of immune cells and the top five principal components extracted from the genotype data were not included in the model.

Multiple testing correction was performed based on the total number of CpGsites in the 250k bp up and downstream of the *SKAP1* gene.

### Genetics

At first genotyping, quality control and imputation was performed. All participants of multiple ethnicities from the 2000HIV cohort were genotyped on the Illumina Infinium Global Screening Array. After quality control, 582 404 variants from 1864 individuals were retained for the imputation procedure. We found 10 810 841 variants from 1864 individuals of the 2000HIV multiancestry cohort. To test whether SNPs influence plasma protein concentrations of SKAP1, protein quantitative trait locus (pQTL) mapping was performed. Thereafter, the association between genetic variants and the INR phenotype was tested using all individuals from European ancestry with known INR status in the 2000HIV cohort. For full description, see Supplemental information 2, Supplemental Digital Content.

### *SKAP1* transcription in HIV infected and non-HIV infected CD4^+^ T cells

CD4^+^ T cells, isolated from PBMC from healthy donors, were HIV infected in vitro or not. Comparison of RNA transcription in HIV-infected an uninfected cells was performed using an un-paired t-test. For complete information, see Supplemental information 3, Supplemental Digital Content.

### Statistical analysis

Varying statistical methods are described per assay. For baseline characteristics, we used Mann Whitney U test for continuous variables and Fisher's exact test for categorical variables. For proteomics and epigenomics, multiple testing was correction was performed in the discovery cohort using the Bonferroni false discovery rate (FDR) method. A value of FDR <0.05 in the discovery cohort and unadjusted P value < 0.05 in the validation cohort was considered significant. Unless specified otherwise all analyses were conducted using R Studio version 4.2.2 (October 31, 2022).

## Results

In the 2000HIV study, we identified 88 INR and 1467 IR, separated into a discovery cohort (62 INR, 1224 IR) and a validation cohort (26 INR, 243 IR), whose baseline characteristics are described in Table 1. In the discovery cohort, INR were significantly older, had a lower CD4 nadir, more often had a history of an AIDS defining disease and more often had a history of a non-AIDS malignancy compared to IR.

**Table 1:** Baseline characteristics of INR and IR in the discovery and validation cohort. Continuous variables were compared using the Mann–Whitney *U* test, categorical variables using Fisher's exact test.

	Discovery cohort			Validation cohort		
	Nonresponder	Responder	*P*-value	Nonresponder	Responder	*P*-value
						
	*n* = 62	*n* = 1,224		*n* = 26	*n* = 243	
Age in years; median (IQR)	55.5 (47.0–63.0)	52.0 (42.8–59.0)	0.017	54.0 (48.5–59.8)	53.0 (47.0–61.0)	0.92
Sex (male)	52 (83.9%)	1,036 (84.6%)	0.86	25 (96.2%)	198 (81.5%)	0.059
BMI in kg/m^2^; median (IQR)	24.8 (22.0–27.6)	24.9 (22.5–27.7)	0.54	25.2 (23.1–26.5)	25.7 (23.0–27.9)	0.62
Age at diagnosis; Median (IQR)	41.5 (30.2–52.2)	35.9 (28.3–43.7)	0.006	40.5 (32.7–47.3)	40.8 (32.8–48.0)	0.87
HIV duration in years (IQR)	12.2 (6.7–18.9)	13.1 (8.2–19.6)	0.3	9.8 (4.8–18.0)	10.9 (5.8–16.7)	0.58
cART duration in years; median (IQR)	11.3 (6.2–16.6)	10.5 (6.5–16.5)	0.96	7.9 (3.9–16.6)	8.7 (5.2––14.1)	0.76
Latest CD4^+^ cell count (×10^6^ cells/l); median (IQR)	300.0 (250.2–330.0)	780.0 (640.0–970.0)	<0.0001	300.0 (262.5–330.0)	740.0 (610.0–905.0)	<0.0001
CD4 Nadir (×10^6^ cells/l); median (IQR)	63.0 (20.0–142.0)	280.0 (180.0–410.0)	<0.0001	100.0 (60.0–150.0)	310.0 (210.0–455.0)	<0.0001
History of AIDS defining disease	27 (43.5%)	215 (17.6%)	<0.0001	11 (42.3%)	39 (16.0%)	0.003
Viral load zenith; median (IQR)	117946.0 (44473.8–284045.0)	100000.0 (37612.0–248855.0)	0.56	457896.0 (135918.2–721310.0)	150000.0 (44054.5–334635.5)	0.006
Currently smoking	16 (25.8%)	376 (30.7%)	0.56	5 (19.2%)	75 (30.9%)	0.35
History of non-AIDS malignancy	9 (14.5%)	42 (3.4%)	0.0004	1 (3.8%)	15 (6.2%)	1.0

*P*-value indicates the significance between nonresponders and responders in the discovery cohort.

cART, combination antiretroviral therapy; IQR, inter quartile range; INR, immunological nonresponders; IR, immunological responders.

### SKAP1 circulating concentrations are significantly lower in INR

**Fig. 1:**
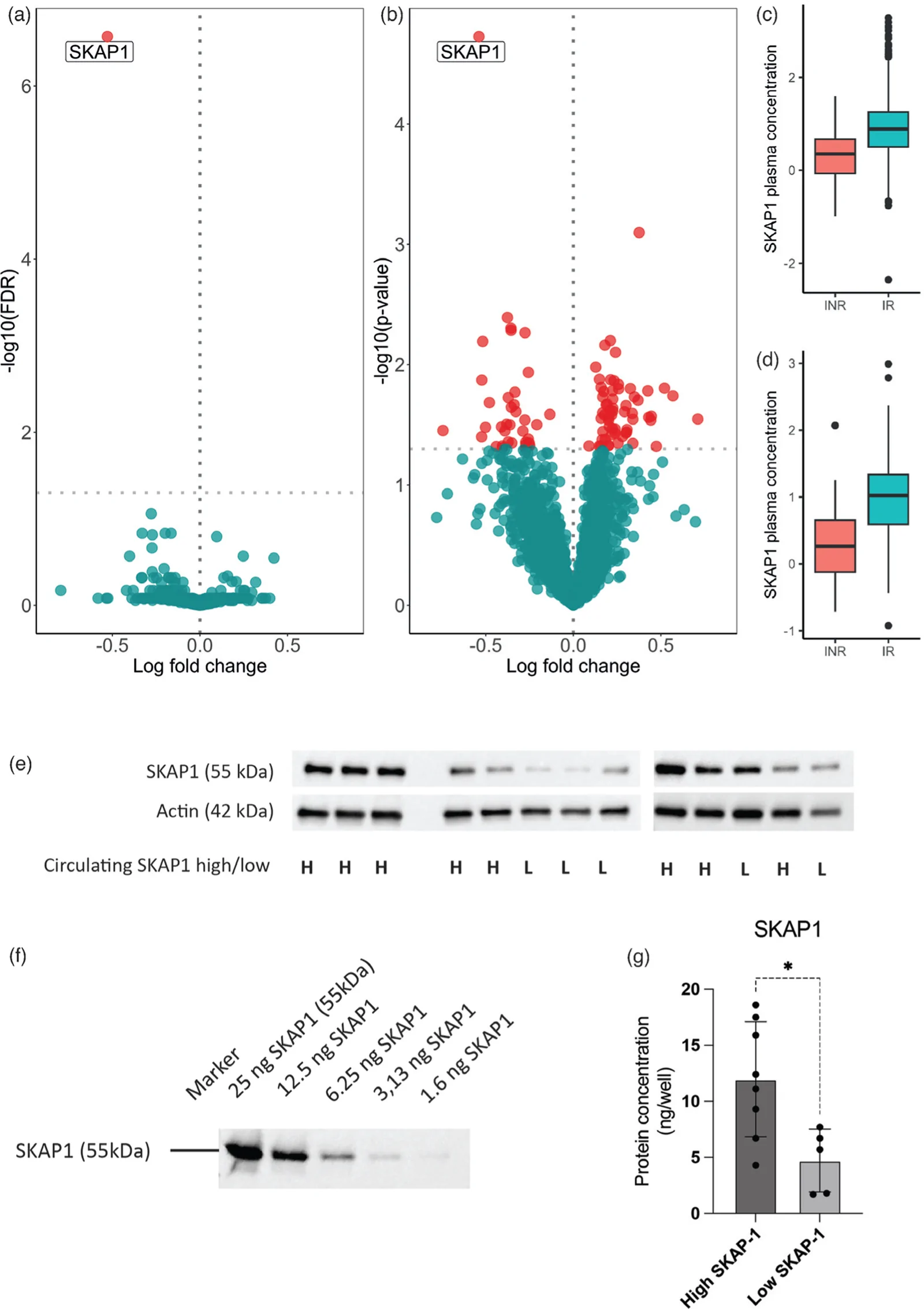
Difference in protein SKAP1 expression concentrations in INR compared to IR.

To identify plasma proteins associated to INR, we compared the concentrations of 2367 plasma proteins between INR and IR. For differential expression (DE) analysis, we used a linear model with age, sex, seasonality, BMI and current smoking status as covariates (Supplementary Figure 1, Supplemental Digital Content). After multiple testing correction, we identified SKAP1 as a significantly differentially expressed biomarker in the discovery cohort (logFC = −0.529, FDR = 2.69 × 10^−7^) that was lower expressed in INR (Fig. 1a, c). This finding was also validated in the second independent validation cohort (logFC = −0.538, nominal *P*-value = 1.88 × 10^−9^) (Fig. 1b, d).

(a, b) Differential expression analysis of plasma protein concentrations in INR compared to IR. *x*-axis shows the size of fold change of proteins, *y*-axis depicts level of significance. Plasma concentrations of 2367 proteins were compared between INR and IR, corrected for sex, age, seasonality, BMI and current smoking status. In the discovery cohort (a) only SKAP1 was significantly (FDR < 0.05) differentially expressed, with a lower expression in INR. In the validation cohort (b) we were able to validate lower concentrations of SKAP1. (c) Comparison of SKAP1 normalized expression values (NPX) concentration in the discovery cohort between INR and IR. (d) Comparison of SKAP1 normalized expression values (NPX) concentration in the validation cohort between INR and IR. (e) Intracellular SKAP1 protein quantification in CD4^+^ T cells stratified based on IR with high (H) and INR with low (L) circulating SKAP1 concentrations as measured in plasma proteome (actin as loading control). (f) Standard curve using rhSKAP1 protein. (g) Relative expression of SKAP1 protein in CD4^+^ T cells corrected for actin expression. The amount of protein/well was calculated using the values obtained from the standard curve. Mann–Whitney *U* test, *P* < 0.05, high (*n* = 8) vs. low (*n* = 5) circulating SKAP1. INR, immunological nonresponders; IR, immunological responders.

SKAP1 is known to be an intracellular protein. Therefore, the intracellular levels of SKAP1 in CD4^+^ T cells were measured to confirm that low SKAP1 plasma levels reflect low intracellular levels. Using Western blotting, we assessed intracellular SKAP1 concentrations in five INR samples that showed the lowest SKAP1 plasma concentrations and in eight IR with the highest SKAP1 plasma concentrations (Figure 1e–g). We observed that IR with the highest plasma SKAP1 had significantly higher intracellular SKAP1 concentrations than INR with lowest plasma SKAP1 (*P*-value < 0.05), confirming that SKAP1 plasma concentrations are reflective of SKAP1 concentrations inside CD4^+^ T cells.

#### INR show increased methylation at the *SKAP1* promotor region

**Fig. 2:**
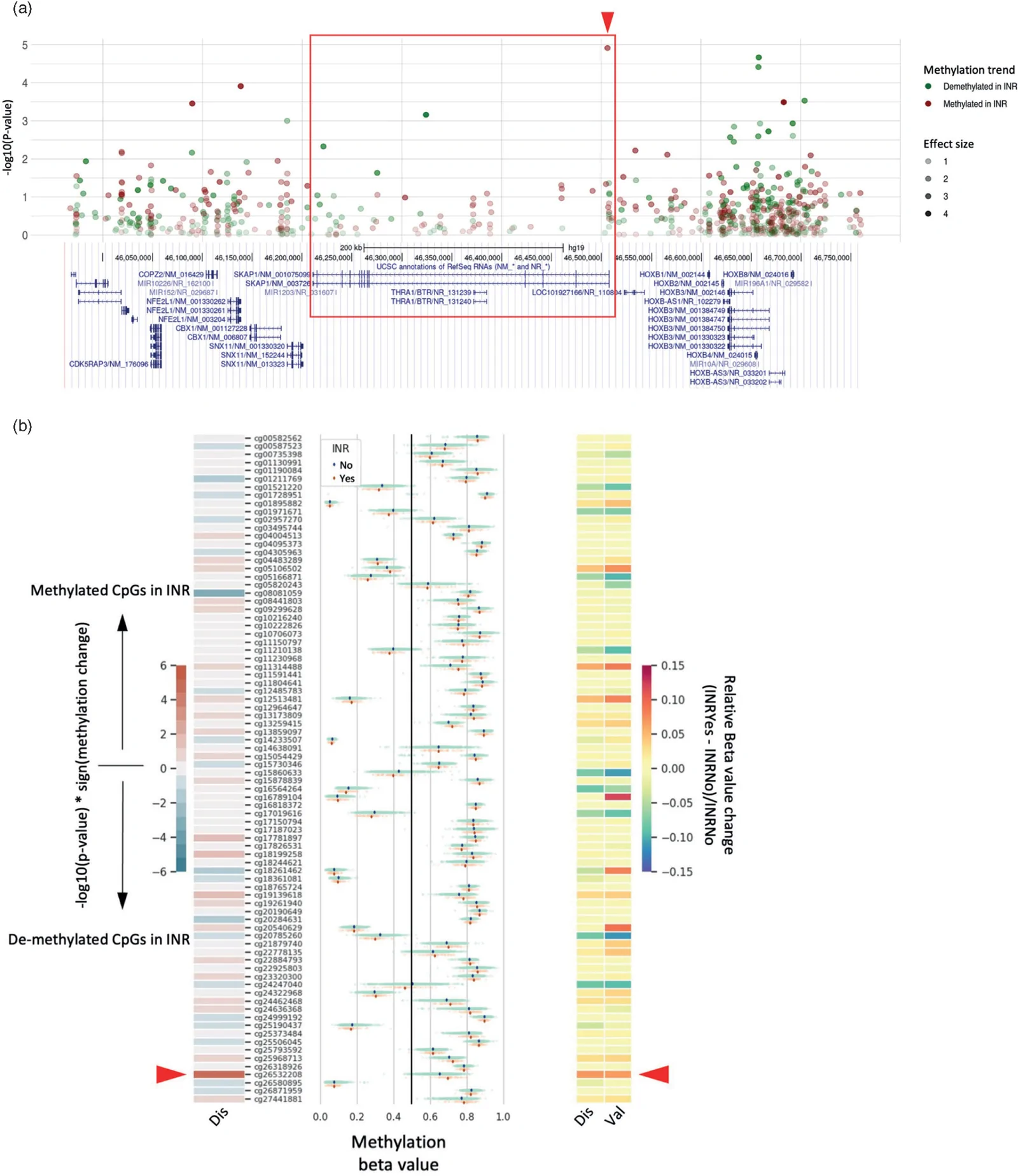
DNA methylation of *SKAP1* gene region.

To investigate whether *SKAP1* downregulation could be the result of DNA methylation changes in the promoter region of the gene, we compared the CpG sites around the *SKAP1* gene (250k bp up- and downstream) between INR and IR. We identified a CpG site with significantly increased methylation levels (cg26532208, Fig. 2 and Supplementary Table 1, Supplemental Digital Content, arrowhead pointed, *P*-value = 1.2e−5, FDR = 0.008) located in the promoter region of *SKAP1* in INR. This difference was also confirmed in the validation cohort (Fig. 2, Supplementary Table 1, Supplemental Digital Content, nominal *P*-value = 0.000114). Furthermore, the absolute DNA methylation change between INR and IR is also higher compared to the other observed CpG sites (Fig. 2, middle and right panel). Increased DNA methylation in the promoter region can recruit Methyl-CpG Binding Proteins, which contribute to the formation of a repressive chromatin structure [19], and it can also inhibit the transcription initiation by preventing the transcription factor binding [20]. All these can lead to long-term silencing or reduction of gene expression. These data suggest that epigenetic mechanisms at the level of DNA methylation may contribute to the decreased SKAP1 concentrations in INR compared to IR.

Methylation at CpG sites in a 500 kb window from the *SKAP1* gene was associated with the INR status using a linear regression model correcting for age, sex, BMI, seasonality, technical covariates and immune cell composition. (a) Regional association plot of the *SKAP1* locus in the discovery cohort. The *x*-axis represents the chromosomal position on hg19, with the lower panel representing genes encoded in the locus. The *y*-axis of the upper panel shows the –log_10_(*P*-value) for association with the INR phenotype. Colors of the dots indicate the beta-value of the association, with red showing hypermethylation in INR and green demethylation in INR. (b) Overview of all CpG sites in the *SKAP1* locus. The left panel shows the strength of the association, the middle panel shows the beta-value in INR and IR and the right panel shows the relative difference between beta values in INR and IR. The red arrows indicate cg26532208. INR, immunological nonresponders; IR, immunological responders.

#### Genetic variation is associated with lower SKAP1 concentrations in PWH

Given the SKAP1 downregulation in INR, we next investigated whether the plasma protein concentration was regulated by genetic variants in the vicinity of *SKAP1* gene (±500 kb). For this, we performed pQTL mapping for SKAP1 plasma concentrations in individuals of European ancestry using the discovery (*n* = 1064) and validation cohort (*n* = 256) (Supplementary Figure 2, Supplemental Digital Content). Furthermore, we tested if the identified *cis*-pQTLs are associated with the INR phenotype using a logistic regression model with age, sex and the first five genetic PCs as confounders using 57 INR vs. 1000 IR of European ancestry.

**Fig. 3:**
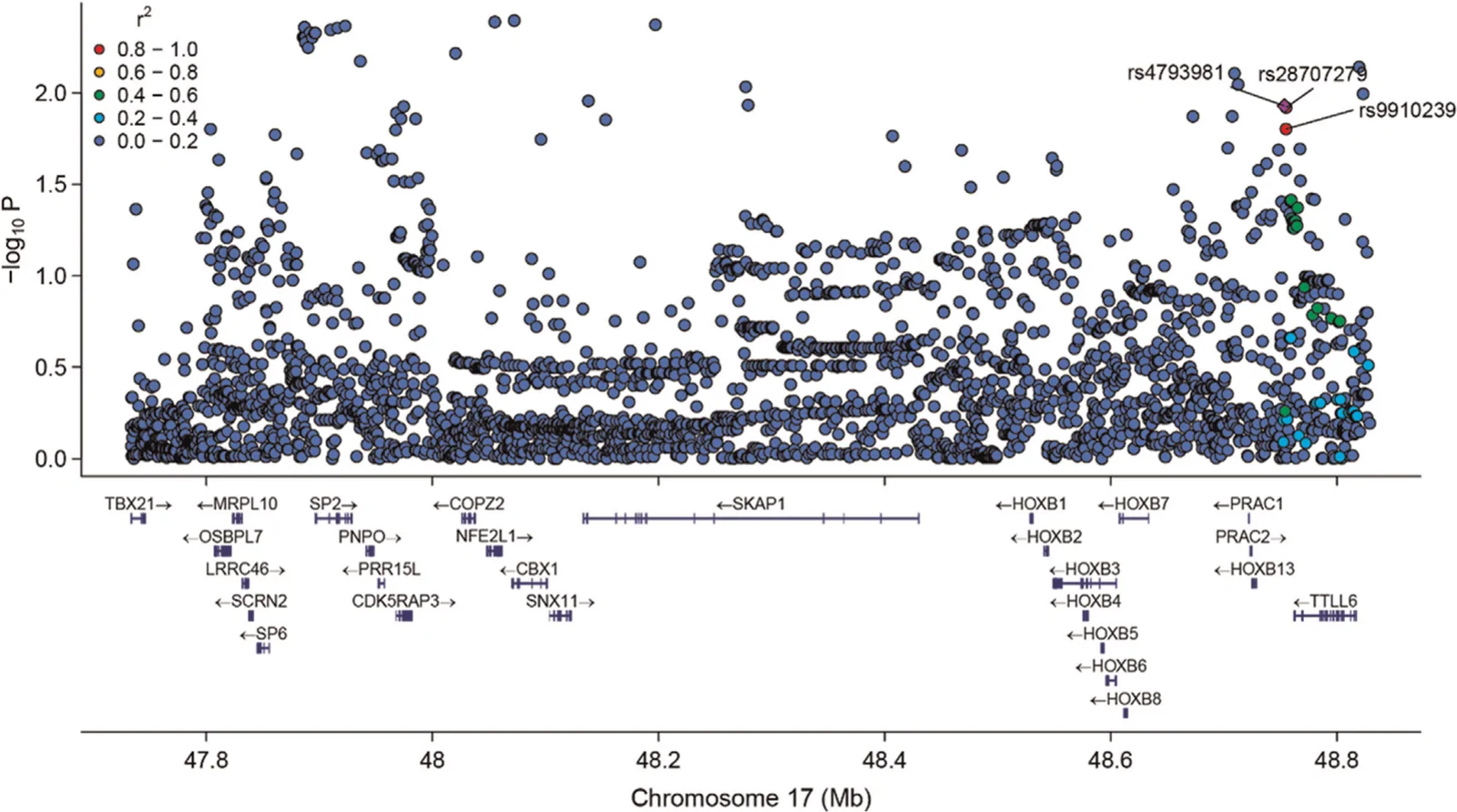
Genetic regulation of SKAP1 protein concentrations in region of 500 kb surrounding SKAP1.

For the discovery cohort, we identified 117 *cis*-pQTLs (*P*-value < 0.05) associated to protein concentrations of SKAP1, of which four could be validated in our validation cohort (Table 2a). These four validated pQTLs (rs4793680, rs4793681, rs4794333, and rs886444) were found to be associated with increased SKAP1 plasma protein concentration but were not associated to the INR phenotype (*P*-value < 0.05, Table 2a). In addition, we identified three genetic variants in the discovery cohort (*P*-value < 0.05; rs4793981-C, rs9910239-G, rs28707279-A) in high linkage disequilibrium (LD), approximately 310 kb upstream of the *SKAP1* gene, which are associated with lower odds of the INR phenotype (OR < 1, Table 2b, Fig. 3). However, these three pQTLs could not be validated in our validation cohort. In addition, their association with lower odds of INR phenotype and decreased SKAP1 protein concentration is opposite to the strong decrease of SKAP1 protein concentration we observed in INR.

**Table 2:** Summary statistics of *cis*-pQTLs for SKAP1 associated with the INR phenotype.

a					SKAP1 pQTL mapping				Association testing INR	
SNP	Chr	Pos	Ref	Alt	*β* discovery	*P*-value discovery	*β* validation	*P*-value validation	OR	*P*-value
rs4793680	17	47852955	A	C	0.090	0.030	0.178	0.037	0.948	0.779
rs4793681	17	47852956	G	A	0.091	0.029	0.179	0.036	0.948	0.779
rs4794333	17	47968129	T	C	0.107	0.013	0.270	0.004	0.965	0.862
rs886444	17	47974545	G	A	0.108	0.012	0.271	0.004	0.967	0.869

b					SKAP1 pQTL mapping				Association testing INR	
SNP	Chr	Pos	Ref	Alt	*β* discovery	*P*-value discovery	*β* validation	*P*-value validation	OR	*P*-value
rs4793981	17	48 753 565	T	C	−0.11	0.012	0.086	0.33	0.64	0.036
rs9910239	17	48 754 972	T	G	−0.11	0.016	0.089	0.32	0.64	0.033
rs28707279	17	48 755 101	G	A	−0.11	0.012	0.089	0.31	0.64	0.035

(a) Validated *cis*-pQTLs for SKAP1 locus and their association with the INR phenotype. *β*-values and OR are given with respect to the alternative allele. (b) *cis*-pQTLs for SKAP1 locus in the discovery cohort and their association with the INR phenotype, *β*-values and OR are given with respect to the alternative allele.

Alt, alternative allele; Chr, chromosome; INR, immunological nonresponder; OR, odds ratio; Pos, position in base-pairs; pQTL, protein-quantitative trait locus; Ref, reference allele.

Regional association plot of the *SKAP1* region and the genetic associations with SKAP1 protein concentration (pQTLs) in people with HIV (PWH). *y*-axis represents the unadjusted –log_10_(*P*-value) and *x*-axis the chromosomal position. SNPs annotated in plot and table were also found to be associated to INR (*P* < 0.05 in the discovery cohort). Color represents LD with rs479381. INR, immunological nonresponders; IR, immunological responders.

Genotype-stratified boxplots of the seven described SNPs in relation to SKAP1 concentrations can be found in Supplementary Figure 3, Supplemental Digital Content.

Taken together, we identified QTLs for SKAP1 protein concentration in close proximity to the *SKAP1* gene in PWH, but these could not be consistently associated with the INR phenotype.

#### HIV infection and CMV coinfection may decrease SKAP1 transcription in CD4^+^ T cells

Finally, we explored factors that could itself could induce changes in SKAP1 expression in CD4^+^ T cells. First, we performed in-vitro HIV infection assays on CD4^+^ T cells from healthy donors. As expected, an increase in the amount of HIV cell-associated RNA at 120 h compared to 48 h postinfection (5.05-fold increase) was observed. Comparing the uninfected and infected samples at the same time points, the gene expression of SKAP1 in infected cells tended to be decreased compared to uninfected cells, although this did not reach the threshold of significance (Supplementary Figure 4, Supplemental Digital Content).

Lastly, we explored the influence of CMV co-infection on SKAP1. We analyzed single-cell RNA transcriptomics data of our cohort and observed in CD4^+^ naïve T-cells that CMV positive participants had lower SKAP1 RNA levels compared to CMV negative participants in (data not shown). We observed a similar trend for activated T-cells (data not shown). Unfortunately this dataset did not contain a sufficient number of INR participants to fully investigate INR.

## Discussion

The present study shows that SKAP1, previously also known as SKAP-55, is the only statistically significant differentially expressed plasma protein in immunological non responders (INR) compared to immunological responders (IR) in two independent cohorts from the 2000HIV study. The decreased plasma concentrations of SKAP1 in INR were in line with the observation of lower intracellular concentrations in CD4^+^ T cells, where SKAP1 is known to exert its function. Furthermore, we show DNA hypermethylation of the *SKAP1* promotor region in INR, indicating that the downregulation of *SKAP1* in INR is epigenetically modified. It is generally accepted that DNA methylation represses gene expression by limiting the binding of transcription factors and/or RNA polymerases to their binding site [21]. Furthermore, we have preliminary data suggesting that HIV itself or CMV co-infection may modulate *SKAP1* expression. Finally, to test whether the decreased SKAP1 plasma protein concentration seen in INR phenotype is genetically regulated, we performed pQTL mapping for SKAP1 concentration and a logistic regression model to test association of pQTLs with the INR phenotype. In the current study, genetic variants associated to INR in close proximity to *SKAP1* gene, could not be validated for their association to SKAP1 protein concentration in our validation cohort.

The immune cell adapter molecule SKAP1 has the capacity to regulate different T cell functions. First, SKAP1 plays a role in ‘inside-out’ signaling, a process in which CD3/T cell receptor engagement on CD4^+^ T cells leads to a conformational change of the integrin lymphocyte function-associated antigen 1 (LFA-1) from closed to active state [22–25]. Activated LFA-1 plays an important role in T cell adhesion, migration and the interaction with antigen-presenting cells (APCs) [25]. Second, antigen bound to activated LFA-1 can induce a process termed “outside-in” signaling resulting in T cell activation, proliferation and differentiation [23,25–27]. Third, SKAP1 was identified as a scaffold for polo-like kinase 1 (PLK1) where it plays a key role in cell cycle progression an proliferation [28,29]. SKAP1 siRNA impedes these processes but can be rescued through the addition of regular SKAP1 [28].

Combined these processes indicate that a lack of SKAP1 may limit the interaction between T cells and antigen presenting cells and impair T cell activation, proliferation and clonal expansion in response to antigen stimulation. IL-2 (important in T cell clonal expansion) and Interferon-gamma production were also noticed to be impaired in SKAP1 deficient activated T cells [30]. We previously observed impaired interferon-gamma and IL-22 response after stimulation of T cells in PBMCs in INR in the 2000HIV study [31].

Our data shows that SKAP1 expression is downregulated in INR, which may eventually lead to reduced LFA-1 activation. Previously it was shown that activated LFA-1 facilitates cell-to-cell spreading of HIV by supporting the viral synapse [32]. A reduction of LFA-1 activation might therefore be beneficial in impairing HIV spread through impaired migration of infected CD4^+^ T cells as well as through impairing the viral synapse. LFA-1 plays a crucial role in T cell migration, and studies in LFA-1 knock-out mice [33] showed lower proliferation capacity and migration to the lymph nodes and intestines, the most important HIV reservoir sites [34,35]. In INR, lower fractions of intestinal CD4^+^ T cells have been observed, which is associated with enterocyte damage [36]. However, our data does not allow definitive conclusions on whether any of these mechanism is present or absent in INR and CD4^+^ T cell reconstitution. We attempted to measure LFA-1 activity in INR but failed because of technical issues.

Finally, we present suggestive data on the possible role of HIV itself on SKAP1 transcription using *ex vivo* experiments. Other factors may be important as well as suggested by a recent clinical trial (ACTG A5383) that showed that Cytomegalovirus (CMV) treatment in INR with CD4< 350 resulted in a significant increase of CD4^+^ cell counts [42]. We therefore analyzed single-cell RNA transcriptomics data of our cohort and observed in CD4^+^ naïve T-cells that CMV positive participants had lower SKAP1 RNA levels compared to CMV negative participants in (data not shown). We observed a similar trend for activated T-cells (data not shown). Unfortunately this dataset did not contain a sufficient number of INR participants to further investigate INR.

Our study has several limitation. We cannot exclude the possibility that SNPs associated with INR were missed and/or are false positives, as a result of the relatively low number of INR. In addition, our cohort may have been too limited to confirm the genetic regulation of the decreased SKAP1 plasma concentration in INR. Another limitation is that our observations are mainly associations and causality cannot be inferred. Also, HIV induced SKAP1 RNA changes as demonstrated in our *ex* vivo experiment showed a trend but did not reach significance and are therefore only suggestive. All study participants were virally suppressed because of cART which inhibits viral replication, however HIV transcripts may still be produced by the latent HIV reservoir and interact with the immune system. Based on our data, no definitive mechanistic connection can be made between HIV infection and reduced SKAP1. The effect of CMV on SKAP1 expression and presence or absence of SKAP1 associated processes (such as LFA-1 activation) should also be further explored.

Overall, we observed low levels of plasma SKAP1 levels in PWH failing to adequately restore CD4^+^ T cell counts despite durable viral suppression. Increased methylation of the SKAP1 promotor suggests that epigenetically regulation may play a role. Given its known role in the function of the immune system, low SKAP1 may play a role in the failure of these individuals to restore CD4^+^ T cells, although a definitive mechanism remains unknown. Potential mechanisms here may be the effect of HIV infection itself or CMV coinfection. SKAP1 is known to regulate T cell migration, proliferation and clonal expansion [28,29]. Low SKAP1 concentrations may therefore contribute to the lower numbers of CD4^+^ T cells in blood and tissues, where the HIV reservoir persists. Overall, this study opens up new opportunities for further mechanistic studies that should elucidate the role of SKAP1 in INR, which could be a potential drug target for the treatment of INR [37–41].

## Acknowledgements

Our gratitude goes to all volunteers who were willing to participate in this study. We thank all the research teams of the 2000HIV study within the Human Functional Genomics Project (HFGP) for their collaboration and support. We thank the Stichting HIV Monitoring for providing us with relevant clinical data from ATHENA cohort: The ATHENA database is managed by Stichting HIV monitoring and supported by a grant from the Dutch Ministry of Health, Welfare and Sport through Centre for Infectious Disease Control of the National Institute for Public Health and the Environment.

Author contributions: W.V., X.J., S.R., N.U., W.S., J.C.d.S., V.M., and N.V. performed the analyses. W.V., S.R., X.J., N.U., W.S., J.C.d.S. and V.M. wrote the manuscript. A.G., M.B., L.E. collected data and assisted in analyses. W.S., J.C.d.S., M.C., H.M. and M.Ö. designed and performed the lab experiments. K.B., W.B., J.St., Y.L., C.X., J.C.d.S., V.M., and A.V. supervised the work. M.N., J.L., and A.V. designed the project. All authors read and approved the manuscript.

Data sharing: All participant data were handled in accordance with applicable data protection regulations.

The olink dataset is available in the Radboud Data Repository (RDR; DOI 10.34973). Genetic and methylome are submitted to the European Genom-Phenome Archive (EGA) but are still under review by EGA. The 2000HIV research group is in the process of publishing the clinical dataset in the RDR and is currently available upon reasonable request.

Funding and declaration of interest: The 2000HIV study was funded by ViiV Healthcare. Although there is close collaboration, ViiV Healthcare did not have any role in data quality control, statistical analyses and final interpretation of the data.

### Conflicts of interest

There are no conflicts of interest.

## Supplementary Material

Supplemental Digital Content
