# Supplementary material for: HIV immunological nonresponders show low SKAP1 concentration and DNA hypermethylation in the SKAP1 promotor region
Source: AIDS. 2026 May 11;40(9):1225–35. doi: 10.1097/QAD.0000000000004529 (PMC13566367; doi:10.1097/QAD.0000000000004529)
Supplement: Supplementary file 1 [file aids-40-1225-s001.docx]

**Supplementary material:**

**Supplementary figure 1.** Principal component analysis on the first 5 principal components of 2367 plasma proteomics to identify confounders of protein concentrations in INR compared to IR. Based on this analysis we corrected plasma protein analysis for sex, age, seasonality, BMI and current smoking status.

 **Supplementary figure 2**. Ancestry principal component analysis (PCA) for genetic association testing. Individuals and single nucleotide polymorphisms (SNPs) that passed quality control (QC) were merged with the 1000genomes reference genomes and PCA was performed in PLINK 1.9b. Colour represent the superpopulations within the 1000genomes, with black dots representing individuals from the 2000HIV cohort. The black rectangle represents the mean +- 3SD from the European reference population on principal component (PC)1 and PC2 and individuals that did not fall within the rectangle were excluded from analysis.

**Supplementary figure 3**: Genotype-stratified boxplots of seven observed SNPs and their relation to SKAP1 plasma concentrations.

**Supplementary figure 4. Expression levels of SKAP1 RNA during infection of CD4+ T cells with HIV.** CD4+ T cells were isolated from four healthy donors and either infected (INF) or not infected(UI) with HIV. Samples were taken 48 and 120 hours post-infection for RNA extraction and subsequent qPCR. The relative expression of HIV cell-associated RNA (A) and SKAP1 expression (B,C) was measured for each sample. The expression of SKAP1 was assessed using two primer pairs, P1 and P2. Each donor is indicated in a different colour. Expression levels were normalized based on the expression of three reference genes (B2M, TBP and YHWAZ). HIV infection-induced decreased production of SKAP1 is confirmed by the analysis with both primers, especially at the 120-hour timepoint.

C

B

A

**Supplementary table 1.** DNA methylation around the SKAP1 gene.

Table will be provided separately due to its size

**Supplemental information 1: Intracellular SKAP 1 measurements**

1.1 Isolation of CD4+ T cells
PBMCs of five INR with the lowest and eight IR with the highest SKAP1 concentrations in the plasma proteomics obtained during the baseline visit of the 2000HIV study were thawed and washed twice using RPMI-1640 (RPMI 1640, Dutch Modification Gibco, supplemented with 2 mM Glutamax, 1 mM sodium pyruvate and 50 µg/mL gentamycin (RPMI+++)) with 20% fetal calf serum (Gibco). Cells were assessed for total cell count and viability using Nexcelom’s Cellometer. CD4+ T cells were enriched using MACS kit (CD4 Microbeads human, positive selection, Miltenyi Biotec), according to the manufacturer’s protocol. The yield was assessed on the Cellometer and purity of the CD4+ T cells using flowcytometry (>97% purity).

1.2. Detecting SKAP1 intracellularly using Western blot
To confirm the presence of SKAP1 intracellularly, 1⋅10^6^ CD4+ T cells were lysed in 80 µL RIPA buffer (Roche) supplemented with cOmplete (Roche) and PhosSTOP (Roche) and incubated for 10 minutes on ice. The lysates were centrifuged at 13000 RPM for 10 minutes and supernatant was used to determine protein concentration with BCA assay (Pierce). The samples were normalized and denatured with Laemmli buffer for 5 minutes at 95°C. For quantification purposes, a serial dilution (1.6-25 ng/well) of human recombinant (rh) SKAP1 (cat #TP308493, AMSBIO, inc.) was performed.
The samples were loaded on a precast 4-15% polyacrylamide gel (Bio-Rad) and electrophoresis was performed on 70 V for 2 hours. After electrophoresis, the samples were transferred to nitrocellulose with Turbo transfer buffer (Bio-Rad - 10% ethanol) and the Trans-Blot Turbo transfer system (Bio-Rad). Subsequently, the membranes were incubated with blocking buffer (5% milk powder in Tris-buffered Saline with 0.1% Tween (TBST)) for 1 hour. After removing the blocking buffer, the membranes were incubated with polyclonal primary antibodies anti-SKAP1 (cat #AF4264, R&D systems, 1:500) and anti-actin (cat #A2066, Sigma-Aldrich, 1:1000) overnight at 4°C. After incubation with the primary antibodies, the membranes were washed three times with TBST for 5 minutes and incubated with secondary antibody swine anti-rabbit IgG-HRP conjugate (cat #P0217, Dako, 1:5000) and rabbit anti-goat IgG-HRP conjugate (cat #P0449, Dako, 1:5000), respectively. Proteins were detected using Clarity Western ECL Substrate (Bio-Rad) according to the manufacturer’s instructions and visualised using the Gel Doc XR+ imaging system (Bio-Rad). Quantitative analysis was performed using Image Lab software (Bio-Rad). Signal intensity of SKAP1 and actin (loading control) were obtained after background correction and further normalised using the loading control. Next, the standard curve was used to determine the concentration of protein per well. **Supplemental information 2: Genetic analysis**

2.1. Genotyping, quality control and imputation
DNA was isolated from whole blood for all participants. All participants of multiple ethnicities from the 2000HIV cohort were genotyped on the Illumina Infinium Global Screening Array. Prior to imputation, quality control for raw variants and samples was performed using PLINK v1.90b ^[37, 38]^. Genetic variants with a call rate genotype missingness of > 5% and those deviating from Hardy-Weinberg equilibrium (HWE) with a P value < 1 ∙ 10^-6^ were excluded from the dataset. HWE exact test was performed using variants stratified by ethnicity. Samples with a call rate of < 97.5% and those that showed a heterozygosity rate that deviated over three standard deviations (SD) from the mean heterozygosity rate per self-reported ethnicity were excluded. Genetic variants that passed QC were lifted from GRCh37 to GRCh38 build using the UCSC liftOver tool (http://genome.ucsc.edu) ^[39]^. Next, strand alignment to the TOPMed reference panel was performed using the TOPMed Freeze5 on genome build GRCh38. For this alignment, we used the McCarthy group tools (https://www.well.ox.ac.uk/~wrayner/tools/).
After quality control, 582,404 variants from 1864 individuals were retained for the imputation procedure. The filtered raw variants were uploaded to the TOPMed Imputation server and imputed against the TOPMed (version r2 on GRCh38) reference panel. The imputed variants were filtered using BCF tools stratified by ethnicity ^[40]^, excluding variants with low imputation quality scores (R2 < 0.3 or ER2 < 0.7) and MAF < 1%. This resulted in 10,810,841 variants from 1864 individuals of the 2000HIV multi-ancestry cohort.

2.2 Plasma protein quantitative trait locus mapping
To test whether SNPs influence plasma protein concentrations of SKAP1, protein quantitative trait locus (pQTL) mapping was performed using the discovery and validation cohort, both of European ancestry. Post-imputation quality control was carried out using PLINK 2.0. All imputed variants were tested if they fail HWE at P < 1⋅10^-12^ and those with a MAF < 1% and R2 < 0.05 were removed. After quality control, 8,944,122 imputed SNP variants were retained for pQTL mapping. Both genotype and plasma concentrations were obtained for a total of 1064 and 256 individuals from the discovery and validation cohort, respectively. SKAP1 plasma protein concentrations were inverse rank transformed to follow a Gaussian distribution. We mapped the inverse-rank transformed plasma concentrations of SKAP1 to genotype data in both discovery and validation cohort using a linear model correcting for age, sex, BMI, seasonality, inclusion before the covid pandemic, covid vaccination and recruitment center. QTL mapping was performed using the MatrixEQTL package ^[41]^ in R version 4.3.0. Focusing on a region 500kb from the start and end of the *SKAP1* gene, SNPs with P < 0.05 in the discovery cohort were considered to show a suggestive association with *SKAP1*. SNPs that additionally had a P < 0.05 in the validation cohort, and a direction concordant with the discovery cohort, were considered to be validated. Regional association plots were made with the *locuszoomr* package in R version 4.3.0.

2.3 Genetic association testing with INR
Given the limited number of INR in our cohort, the association between genetic variants and the INR phenotype was tested using all individuals from European ancestry with known INR status in the 2000HIV cohort (n = 57). As population stratification is known to be an important confounder, only individuals of European ancestry were included. Post-imputation quality control was carried out using PLINK 1.90b. First, indels and copy number variants, non-autosomal variants, variants that failed HWE (P < 1 ⋅ 10^-12^) and those with MAF < 5% were removed. Additionally, individuals with outlying heterozygosity (those that deviate three SD from the mean heterozygosity rate), discordant sex information (X chromosomal inbreeding coefficient > 0.2 for females or < 0.8 for men), with one sample identified as having Klinefelter syndrome, as well as related individuals (based on the identity-by-descent, IBD > 0.1875) were excluded. Ancestral outliers were detected by PCA (Supplemental fig 2). After quality control, 5,791,803 imputed genetic variants from 1057 samples were retained for follow-up analyses. In this study, we tested whether imputed variants around *SKAP1* and those influencing SKAP1 protein concentrations were associated with INR phenotype using 57 INR and 1000 IR. An additive logistic regression model was used in which age, sex and the first five genetic PCs, to correct for residual population stratification, were included as confounders. SNPs with a P < 0.05 were considered to show a suggestive association with INR phenotype.

**Supplemental information 3: *SKAP1* transcription in HIV infected and non-HIV infected CD4+ T cells**
Human peripheral blood mononuclear cells (PBMC) from healthy donors were isolated by Ficoll density gradients separation (Serumwerk). Isolation of CD4+ T cells was done by positive selection using Human CD4 Microbeads (Miltenyi Biotech) following the manufacturer’s protocol. In brief, PBMCs were incubated for 15 minutes with 20µL of CD4 Microbeads per 10 Million cells in 100µL PBS (Gibco), 2mM EDTA (Invitrogen), and 1% FCS (HyClone) at 4 °C. Cells were then washed with 1.5 mL PBS per 10 million PBMCs and resuspended in 500 µL per 100 million PBMCs. The suspension was put on an LS Column (Miltenyi Biotech), which was loaded on a MACS MultiStand (Miltenyi Biotech). Columns were washed thrice with 3mL PBS, 2mM EDTA, and 5% FCS, and cells were subsequently eluted in 5mL. Isolated CD4+ T cells were cultured in RPMI (Gibco), with 5% Human AB Serum (MP Biomedicals), 1% Pen/Strep (Gibco), and 20 ng/mL recombinant human IL-2 (PeproTech), and activated with 10 µL/mL T Cell Transact™ (Miltenyi) 2 days prior to use in downstream experiments.
Next, the following reagent was obtained through the NIH HIV Reagent Program, Division of AIDS, NIAID, NIH: HIV p89.6 (ARP-3552) (1). For the production of HIV viruses, plasmids were transfected into HEK293T cells using jetOPTIMUS® DNA Transfection Reagent (Polyplus) following the manufacturer’s protocol. In brief, one day prior to transfection, HEK293T cells were seeded at a density leading to 75% confluency. The next day, a dilution of p89.6 plasmid was made at a concentration of 3.3 µg for transfection in a T25 flask. To the plasmid dilution, up to 500 µL jetOptimus buffer was added, and jetOptimus reagent equal to the amount of µg DNA in the mix (e.g., 10 µg is 10 µL). The mixture was gently added to the cells, and after 4 hours, the media were refreshed. Three days post-transfection, viruses were harvested and concentrated 10x using Amicon Ultra-15 (Merck). The HIV 89.6 virus stock was titrated using the QuickTiter Lentivirus Titer kit (Bio-Connect), following the manufacturer’s manual.
Subsequently, isolated CD4+ T cells were plated out at a 1.6 million/mL density in a 6-well plate at a final cell amount of 2 million. Cells were then infected using HIV 89.6 at the multiplication of infection (MOI) of 1. During the infection of the cells, the media was supplemented with 8 µg/mL polybrene (Merck). After adding the virus and polybrene, cells were spinoculated at 900 g for 90 min. Two hours post-spinoculation, the media were changed. Cells were collected 48 and 120 hours post-infection, after which RNA was extracted.
RNA was extracted following manufacturers’ protocols using the innuPREP RNA mini kit 2.0 (Analytikjena). RNA was eluted in 50 µL of RNAse-free water, and concentrations were measured on the Nanodrop 2000 (Thermo Scientific). From the isolated RNA, cDNA was made with the qScript cDNA Supermix (Quantabio). For this, 200 ng of RNA was used for each reaction, to which 4 µL of qScript Supermix was added in a total volume of 20 µL. Reactions were placed in a PCR machine, and the following program was run to convert RNA to cDNA: 5 minutes at 25 °C, 30 minutes at 42 °C, 5 minutes at 85 °C and hold at 4 °C. From the samples, 2 µL was used in subsequent qPCR. qPCR reactions were performed in a total volume of 5 µL containing 2 µL of cDNA, 2.5 µL of LightCycler® 480 SYBR Green I Master (Roche Life Science) and forward and reverse primers at a concentration of 250 nM. Each reaction was performed in duplicate. qPCR was run on a LightCycler480 (Roche) with the following cycling program; 5 minutes at 95 °C, followed by 45 cycles of; 15 seconds at 95 °C, 30 seconds at 60 °C and 15 seconds at 72 °C, melting curve from 60 to 95 °C and cooldown at 40 °C for 1 minute. Five reference genes were selected, of which the three most stable were selected with the geNorm method of Vandesomple et al. 2002 (B2M, TBP and YHWAZ) (2). By using the expression of these reference genes, normalization factors were calculated using the qbasePLUS software 3.1 (Biogazelle) via the geometric means of their relative quantities. The normalization factors were then used to calculate the relative expression of *SKAP1* in the samples. The primers used. in HIV infectivity and SKAP1 transcription assay are outlined below.

| **Primer name** | **Sequence** |
| --- | --- |
| B2M Fw | AGATGAGTATGCCTGCCGTGTGAA |
| B2M Rv | TGCTGCTTACATGTCTCGATCCCA |
| TBP Fw | CAAGCGGTTTGCTGCGGTAATCAT |
| TBP Rv | TGCCAGTCTGGACTGTTCTTCACT |
| YHWAZ Fw | ACTTTTGGTACATTGTGGCTTCAA |
| YHWAZ Rv | CCGCCAGGACAAACCAGTAT |
| SKAP1 F1 | GGCAGAAGGTTTGCGGAATG |
| SKAP1 R1 | CCTTGGGGCTGAAAATCCCA |
| SKAP1 F2 | CCCAGTGCAGACCCACTATC |
| SKAP1 R2 | ACTCCTTTTCGTCGAGTGCC |
| HIV-1 LTR Fw | TTAAGCCTCAATAAAGCTTGCC |
| HIV-1 LTR Rv | GTTCGGGCGCCACTGCTAGA |

1.	Collman R, Balliet JW, Gregory SA, Friedman H, Kolson DL, Nathanson N, et al. An infectious molecular clone of an unusual macrophage-tropic and highly cytopathic strain of human immunodeficiency virus type 1. J Virol. 1992;66(12):7517-21.

2.	Vandesompele J, De Preter K, Pattyn F, Poppe B, Van Roy N, De Paepe A, et al. Accurate normalization of real-time quantitative RT-PCR data by geometric averaging of multiple internal control genes. Genome Biol. 2002;3(7):RESEARCH0034.
